# Effect of Posthospitalization Sleep Regularity on Clinical Events in Adults With Heart Failure

**DOI:** 10.1016/j.jacadv.2025.102109

**Published:** 2025-08-21

**Authors:** Brooke M. Shafer, Shirin O. Hiatt, Sophia A. Kogan, Christopher S. Lee, Nathan F. Dieckmann, Christopher V. Chien, Quin E. Denfeld, Andrew W. McHill

**Affiliations:** a Sleep, Chronobiology, and Health Laboratory, School of Nursing, Oregon Health & Science University, Portland, Oregon, USA; b School of Nursing, Oregon Health & Science University, Portland, Oregon, USA; c Boston College William F. Connell School of Nursing, Chestnut Hill, Massachusetts, USA; d Knight Cardiovascular Institute, Oregon Health & Science University, Portland, Oregon, USA; e Oregon Institute of Occupational Health Sciences, Oregon Health & Science University, Portland, Oregon, USA

**Keywords:** cardiovascular disease, hospitalization, Sleep Regularity Index

**Figure:**
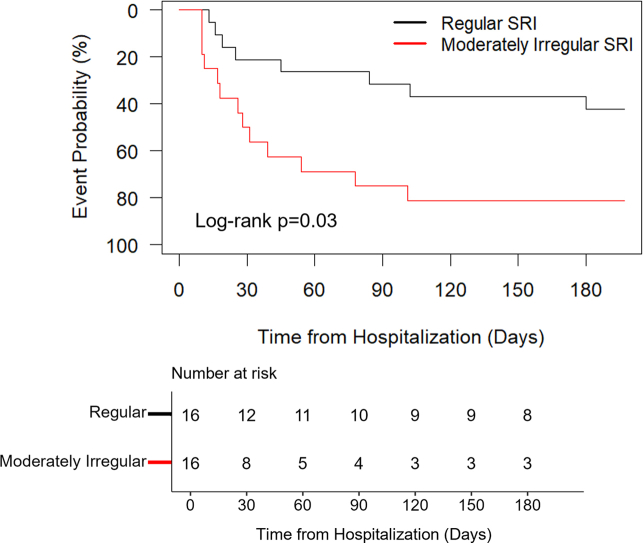

Heart failure (HF) is the leading cause of repeated hospitalization among older adults. Hospital admission for acutely decompensated HF is a sentinel event with a high risk of subsequent readmission or mortality in the vulnerable transition period after hospitalization.^1^ Identifying specific and potentially modifiable contributors to future clinical events is critical for improving clinical outcomes. Day-to-day variations in sleep timing (eg, sleep regularity) may be one novel mechanistic contributor to clinical event risk, as irregular sleep timing is associated with cardiovascular disease incidence.^2^ Furthermore, sleep regularity has been shown to be a greater predictor in overall mortality than sleep duration in the general population.^3^ Adults with HF often report sleep difficulties and less sleep regularity,^1^ thus potentially increasing the adverse event risk. Quantifying the impact of sleep regularity on the 6-month clinical event risk among adults with HF is the key to identifying potential nonpharmacologic behavioral intervention targets to minimize the readmission risk following HF hospitalization.

**What is the clinical question being addressed?**

Does sleep timing regularity predict the 6-month clinical event risk in adults with heart failure?

**What is the main finding?**

Following hospital admission for acutely decompensated heart failure, the clinical event risk was more than double in adults with moderately irregular sleep timing compared to regularly timed sleepers.

## Methods

This secondary analysis included a subset of participant data collected from a longitudinal study described previously.^4^ Briefly, 32 participants (14 females) with a confirmed HF diagnosis of ≥3 months were recruited following acute decompensated HF hospitalization. Demographic and clinical data (eg, HF type, medications) were collected at baseline during hospitalization using surveys and electronic medical records. A Seattle Heart Failure Model (SHFM) score was calculated to estimate HF severity and the Charlson Comorbidity Index (CCI) weighted score was used to assess comorbidities, as described elsewhere.^4^ Following hospital discharge, the Sleep Regularity Index (SRI) was used to quantify the percent probability of a participant being awake or asleep at any 2 time points, 24-h apart and calculated from diary-determined sleep onset and offset (including daytime napping) collected over ∼7 days.^5^ Clinical event data (eg, all-cause emergency room visit, hospitalization, death) were collected through 6 months posthospitalization from electronic medical records. This study was approved by the Institutional Review Board and all participants provided written informed consent.

Participants were classified into regular (SRI>87%) or moderately irregular (SRI<=87%) SRI groups at baseline based on literature demonstrating moderately irregular sleep patterns are associated with an adverse health risk.^5^ Differences between SRI groups were assessed using independent t-tests and chi-square tests. The first event following HF hospitalization was used to generate Kaplan-Meier time-to-event curves between SRI groups and then compared using the log-rank test. Each participant was only counted once. HRs and 95% CIs were estimated by Cox regression using unadjusted and adjusted models controlling for SHFM, CCI score, and sleep apnea.

## Results

Participants were 62.6 ± 15.5 years, had a body mass index of 32.8 ± 9.9 kg/m^2^, had a HF for 6.2 ± 4.5 yrs (n = 26), and a left ventricular ejection fraction of 40.1% ± 17.6% (n = 31). In this sample, 75% of participants were non-Hispanic White, 56% had HF with reduced ejection fraction, 81% had a nonischemic etiology, 50% had type 2 diabetes and stage 3 chronic kidney disease, and 69% had obstructive sleep apnea. Average SHFM was 81% ± 15%. There were no statistically significant differences in demographic or clinical characteristics between SRI groups (all *P* > 0.11).

**Figure 1:**
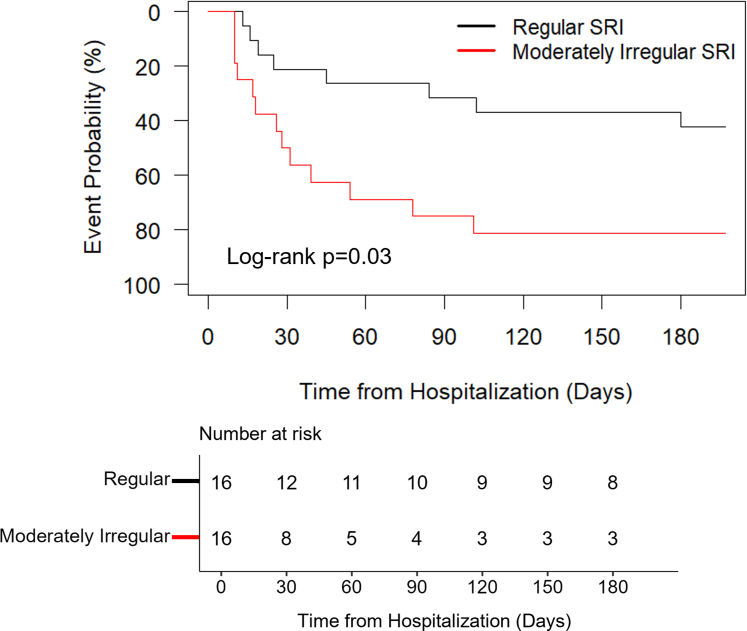
**Kaplan-Meier Time-to-Event Curves** Clinical event risk was compared between moderately irregular (n = 16) and regular (n = 16) SRI groups in adults with HF. Individuals with moderately irregular SRIs (red) had a significantly greater risk of a clinical event compared to regular SRIs (black). *P* value derived from the log-rank test. SRI = Sleep Regularity Index.

Across 6-months, 21 of the 32 participants experienced a clinical event following baseline hospitalization (n = 13 moderately irregular vs n = 8 regular SRI group). Median (Q1, Q3) time to first event was 26 (11,39) days in the moderately irregular SRI group and 35 (18,89) days in the regular SRI group. Compared to regularly timed sleepers, participants with moderately irregular SRIs had significantly higher CCI scores (4.9 ± 1.9 vs 3.4 ± 1.6, respectively; *P* = 0.02) and lower SRIs (79 ± 7 vs 92 ± 3%; *P* < 0.01). Sleep characteristics were similar between SRI groups for Patient-Reported Outcomes Measurement Information System sleep-related impairment (0.32 ± 1.02 vs 0.40 ± 0.78, respectively; *P* = 0.81), sleep duration (581 ± 145 vs 535 ± 83 min; *P* = 0.28), and sleep onset (23:25 ± 1:27 vs 23:12 ± 0:50 clock h; *P* = 0.65). Participants with moderately irregular SRIs had a greater risk of an event (unadjusted: HR: 2.6; 95% CI: 1.1-6.3; *P* = 0.04) compared to those with regular SRIs (Figure 1), which remained significant when controlling for SHFM, CCI score, and sleep apnea (adjusted HR: 3.7; 95% CI: 1.2-12.2; *P* = 0.03).

## Discussion

In this sample of patients recently hospitalized for HF, moderately irregular sleep timing not only predicted the worse 6-month clinical event risk, but the event risk was more than double compared to regularly timed sleepers. These findings highlight the importance of considering overall sleep health when assessing prognostic outcomes among patients with HF, beyond merely addressing sleep-disordered breathing. Sleep disruptions mediated by even moderately irregular sleep timing may be one mechanism underlying the increased risk of rehospitalization and could represent a unique risk factor for future cardiovascular events in HF patients with existing comorbidities.^2^ Understanding the multiple facets of sleep, including regularity, is critical for improving health outcomes for all patients with HF and not just those with sleep disorders.

The week after hospitalization for HF is a particularly vulnerable timeframe as patients are adjusting back to their home environment and re-equilibrating self-care strategies. Factors such as poor sleep during hospitalization and navigating complex medical regimens following discharge may negatively impact the maintenance of a consistent sleep schedule. Guidelines recommend an early posthospitalization follow-up, generally within 7 days, to optimize care and reduce the rehospitalization risk.^1^ Asking about sleep habits during this follow-up may ascertain how well patients are sleeping and identify the potential risk for adverse outcomes.

A few limitations should be considered when interpreting these findings. First, the small sample size limited the capacity to adequately control for multiple potential confounding factors including the treatment status of sleep apnea. Moreover, replication across ethnically diverse cohorts is warranted. Future studies with larger samples reflective of the broader HF population are needed to: 1) confirm these findings; 2) identify how sleep regularity is involved in mechanistic pathways affecting HF outcomes; and 3) uncover how sleep regularity interventions could ultimately inform practice changing recommendations.

This study increases our understanding of how sleep regularity impacts the 6-month clinical event risk in patients hospitalized for HF. Improving sleep regularity may be a low-cost therapeutic approach to mitigate adverse events in adults with HF.

## Funding support and author disclosures

This study recieved funding from 10.13039/100000002NIH T32HL083808, K12AR084221, R01NR019054, OHSU School of Nursing. Dr McHill reports consulting for Pure Somni Corporation and Portland Public Schools. All other authors have reported that they have no relationships relevant to the contents of this paper to disclose.
